# In Vitro Micropropagation of *Rosa canina* L.: From Establishment to Plant Acclimatization

**DOI:** 10.3390/plants15091285

**Published:** 2026-04-22

**Authors:** Maria Casanovas, Emma Roca, Ramon Dolcet-Sanjuan

**Affiliations:** 1 IRTA, Fruitcentre, Plant In Vitro Culture Laboratory, Fruticulture Program, Parc AgroBiotech, 25003 Lleida, Spain; maria.casanovas@irta.cat; 2 Rosehip and Associates S.L., Centelles, 08540 Barcelona, Spain

**Keywords:** dog rose, adventitious rooting, photoperiod, activated charcoal, clonal propagation, vermiculite, acclimatization

## Abstract

*Rosa canina* L. is a medicinal and nutritionally valuable species with increasing industrial demand, yet its conventional propagation is limited by low rooting capacity and high genetic heterogeneity. In this study, a complete and reproducible in vitro micropropagation protocol was established, from explant introduction to plantlet acclimatization. Axillary buds were disinfected and introduced into Murashige and Skoog (MS) medium supplemented with 6-benzylaminopurine (BAP). Shoot multiplication was achieved using sequential cytokinin treatments, and shoot elongation was promoted by adding liquid MS medium containing activated charcoal (AC). The highest and fastest root induction percentage (up to 75%) was obtained on WPM with 2 mg·L^−1^ IBA and under a 16 h light/8 h dark photoperiod. Light promoted adventitious root formation depending on the nutrient formulation. Thereafter, shoots developed well-structured root systems in vitro, and plantlets fully survived to ex vitro acclimatization. This protocol provides an efficient platform for the large-scale propagation of *R. canina* and demonstrates that its auxin-driven adventitious rooting is strongly conditioned by the interaction between basal medium composition and photoperiod.

## 1. Introduction

*Rosa canina* L. (dog rose) is a perennial woody species well known for its medicinal, nutritional, and cosmetic value. All parts of the plant possess valuable bioactive compounds, making it of interest to the pharmaceutical, cosmetic, and food industries: leaves and petals are rich in antioxidant molecules [1,2,3] and the oil extracted from the seeds, traditionally used in cosmetics [4], is currently being investigated for its potential to prevent hepatic steatosis and mitigate metabolic disorders associated with high-fat diets [5,6,7]. Traditionally, *R. canina* has been used in the preparation of juices, jams, marmalades, and syrups [8,9], but more recently, it has gained attention as a source of natural carotenoids and antioxidants for use in food preservation, including meat products [10,11,12,13].

Given the increasing demand for *R. canina* due to its diverse applications, the development of efficient propagation systems is essential to ensure a sustainable and standardized supply of plant material. However, in the present study, conventional propagation through seeds is limited by both endogenous and exogenous dormancy, as well as high levels of abscisic acid and pronounced genetic heterogeneity, which prevents true-to-type reproduction [14,15]. Although vegetative propagation by cuttings is an alternative, many *Rosa* species, including *R. canina*, are difficult to root, often exhibiting low rooting success [16,17].

In this context, in vitro micropropagation via single-node culture offers a powerful tool to overcome these limitations, enabling a reliable and efficient method for the clonal multiplication of *R. canina.* This technique enables the rapid clonal multiplication of genetically uniform, pathogen-free plant material under controlled conditions. Several protocols have been reported for different *Rosa* species, focusing on shoot proliferation using cytokinins, gibberelins, or auxins [18,19,20,21]. However, despite these advances, the rooting phase remains a critical bottleneck in woody plant micropropagation, including *Rosa*, due to its strong dependence on multiple interacting factors such as basal medium composition, plant growth regulators, and environmental conditions. Adventitious root formation in woody species is a complex developmental process regulated by both physiological and molecular mechanisms. Root induction, initiation, and emergence are strongly controlled by auxin, whose biosynthesis, transport, and signaling pathways coordinate the cellular reprogramming required for rooting [22]. In addition to hormonal regulation, light acts as a key environmental signal influencing root formation through photoreceptors, which interact with auxin-related pathways and modulate developmental responses in woody plants [23]. Experimental evidence has shown that light conditions strongly affect the formation of adventitious roots, and certain species cultured in vitro such as *Arabidopsis* and *Vitis* are capable of generating de novo roots under photoperiods with extended darkness [24]. Culture medium composition strongly influences shoot responsiveness to auxin-induced root formation, since variations in nitrogen, phosphorus, or potassium can modulate auxin-mediated regulation of adventitious root initiation [25]. Hence, basal culture medium should be considered since it is expected to affect auxin-driven root initiation. Within this broader framework, several studies on *Rosa* have focused on its known rooting difficulties and point to factors such as the lignin and hemicellulose composition of cell walls [26], as well as the interaction of additional hormonal compounds, such as jasmonic acid and abscisic acid, with auxin [27], adding further complexity to the adventitious rooting process in this genus. These findings support the relevance of evaluating the combined effects of medium composition, auxin, and photoperiod in rooting protocols, particularly in species traditionally considered difficult to root.

The objective of this study was to establish a complete and reproducible in vitro micropropagation protocol for *R. canina*, from explant introduction to plantlet acclimatization, and to experimentally evaluate the effects of (1) basal medium composition, MS medium with a high nitrogen concentration (NH_4_^+^ and NO_3_^−^) vs. WPM medium with lower macronutrient levels and reduced ionic strength, (2) indole-3-butyric acid (IBA) supplementation, and (3) photoperiod or darkness on adventitious root induction and development. By dissecting the individual and combined contributions of these factors, this work provides new insights into the physiological regulation of rooting in woody species and proposes an optimized protocol suitable for large-scale clonal propagation of *R. canina*.

## 2. Results and Discussion

### 2.1. Plant Introduction

The initial phase of in vitro establishment for *Rosa canina* focused on identifying and discarding contaminated or non-viable explants, a critical step for ensuring the success of subsequent developmental stages. A major limitation observed during this phase was the exudation of phenolic compounds, particularly at the basal region of the explants. These compounds, released as a stress response, led to oxidative browning and tissue damage, negatively affecting shoot development.

Phenolic exudation is a common issue in early in vitro culture, often triggered by the activation of plant defense mechanisms and polyphenol overproduction. Leakage of these compounds into the medium is typically associated with cell death [28], and their accumulation can inhibit morphogenesis and growth. To mitigate these effects, explants were regularly transferred to fresh medium, a strategy previously reported as effective [29]. In the present study, weekly excision of the basal portion and medium renewal proved successful in reducing phenolic accumulation and promoting bud sprouting and shoot elongation. These results highlight the importance of early intervention and individualized culture conditions during the in vitro establishment phase.

### 2.2. Shoot Propagation and Elongation

**Figure 1:**
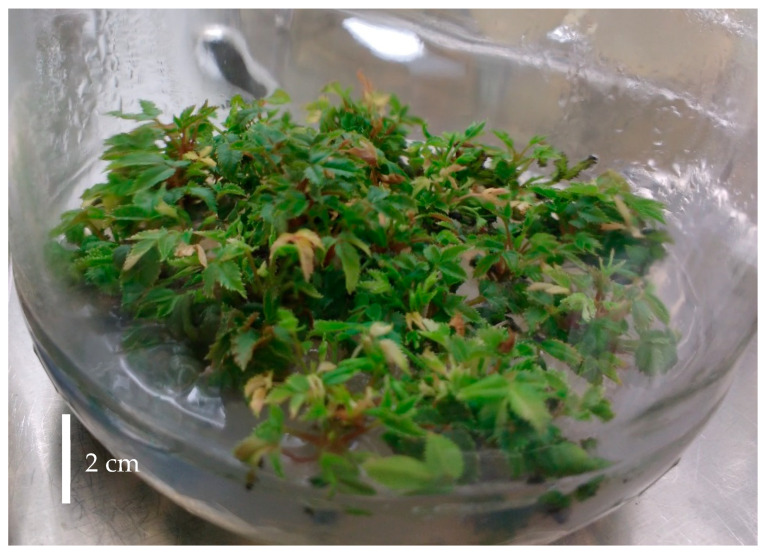
Shoots of *Rosa canina* after 3 weeks of culture in MS medium with 2 mg·L^−1^ BAP.

Shoot multiplication was conducted in two sequential phases using 6-Benzylaminopurine (BAP) as the sole plant growth regulator. In the first phase, newly developed shoots were cultured on Murashige and Skoog (MS) medium supplemented with 2 mg·L^−1^ BAP, resulting in a high proliferation rate of axillary shoots after 3 weeks; however, shoot elongation was limited, presumably due to the elevated cytokinin concentration (Figure 1).

At this point, to promote elongation, shoots were transferred to MS medium containing a reduced BAP concentration (1 mg·L^−1^) and cultured at lower shoot density (8 shoots per jar vs. 12 shoots per jar in the previous step). Under these conditions, compact shoots elongated modestly (5–8 mm). As elongation remained insufficient for rooting, a third phase was introduced by adding a liquid MS medium devoid of plant growth regulators and supplemented with 3 g·L^−1^ activated charcoal (AC).

**Figure 2:**
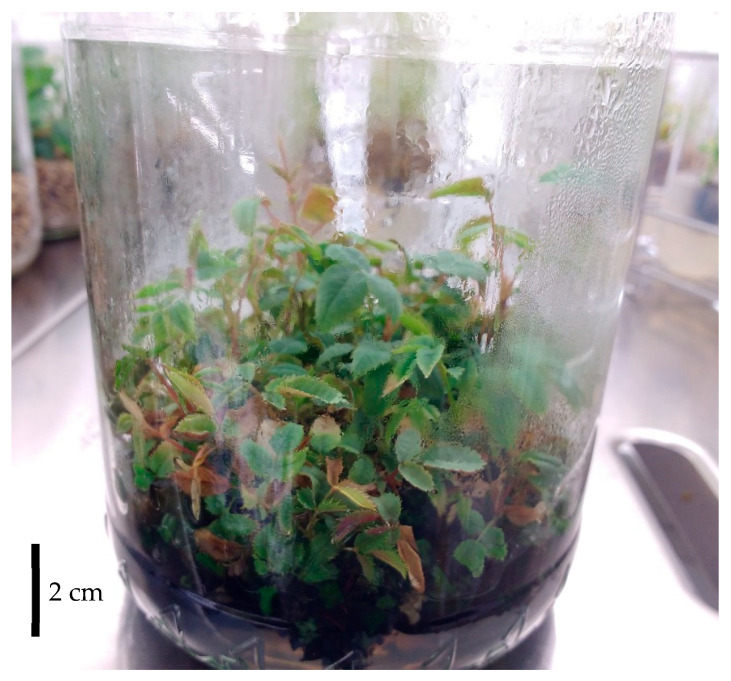
Shoots of *Rosa canina* after 3 weeks of culture with liquid elongation medium (MS + 3 g L^−1^ AC).

AC is commonly employed in in vitro culture systems for its capacity to adsorb excess PGRs and other inhibitory compounds in both liquid and solid media, thereby promoting shoot elongation and physiological development [30,31,32]. In this context, the adsorption of residual cytokinins from the previously used medium by the activated charcoal facilitated shoot elongation after an additional 3-week period (Figure 2).

Several studies have explored the use of various combinations of cytokinins, auxins, and other growth regulators—such as gibberellic acid (GA_3_)—to enhance shoot multiplication in *Rosa* species [18,19,20]. In a more recent study, Pahnekolayi et al. [21] optimized the micropropagation protocol for *R. canina* and *Rosa beggeriana*. These authors evaluated 20 treatment combinations involving BAP (0–2 mg·L^−1^), GA_3_ (0–0.5 mg·L^−1^), and naphthaleneacetic acid (NAA) (0–0.1 mg·L^−1^) and reported that the highest number of axillary shoots and new leaves in *R. canina* was achieved using MS medium supplemented solely with 2 mg·L^−1^ BAP, without GA_3_ or NAA.

**Figure 3:**
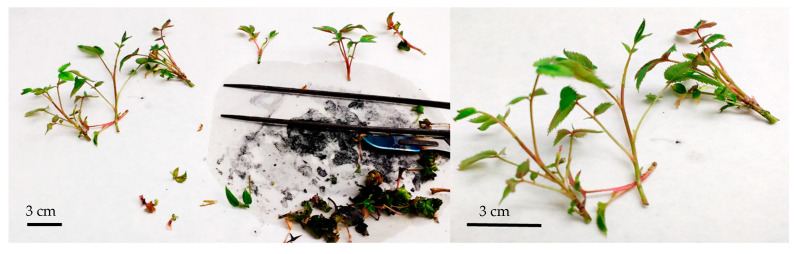
Elongated *Rosa canina* shoots after 3 weeks of culture in a two-phase culture medium, with liquid elongation medium (MS + 3 g L^−1^ AC).

Herein, BAP was likewise used as the sole plant growth regulator. However, unlike the aforementioned protocol, which combined multiplication and elongation in a single step, our approach separated these stages into two distinct multiplication phases followed by an elongation phase prior to root induction. This sequential strategy resulted in a high number of *R. canina* shoots with sufficient elongation (≥3 cm), enabling successful transition to the rooting phase. *R. canina* shoots obtained after the multiplication and elongation phases (Figure 3) were optimal for being transferred to the rooting induction medium.

### 2.3. Root Induction

Root induction in *R. canina* was optimized through two sequential assays designed to unravel the effects of basal medium composition, activated charcoal (AC), auxin supplementation (IBA), and photoperiod on adventitious root formation.

Woody Plant Medium (WPM) was initially selected for the first assay because it was specifically formulated for woody species [33] and is characterized by a lower ionic strength than Murashige and Skoog (MS) medium, reducing osmotic stress and improving explant performance during rhizogenesis. Given that *R. canina* is considered a difficult-to-root species, MS medium was subsequently included in the second assay as a reference medium to assess whether rooting efficiency was primarily driven by salt composition or by the interaction between mineral nutrition, auxin, and light conditions. IBA and AC were evaluated separately to avoid confounding effects, as AC can adsorb phenolic compounds and residual cytokinins, improving the rooting environment, whereas IBA directly promotes root initiation by activating auxin-responsive pathways.

#### 2.3.1. Assay 1: Effect of AC

In the first assay, accumulative rooting percentages were recorded on shoots cultured on WPM basal medium without auxin under a 16 h light/8 h dark photoperiod, comparing basal medium without AC (T1) and WPM supplemented with 3 g·L^−1^ AC (T2). Mean values and their corresponding standard errors (SE) are shown in Table 1.

During the first six weeks of culture, rooting percentages increased progressively in both treatments, being consistently higher in the AC-free treatment (T1). However, at week 7, the AC treatment exhibited an abrupt increase, culminating in significantly higher final rooting by week 8 (58.3% in T2 vs. 41.7% in T1).

Statistical analysis revealed a highly significant effect of time (*p* < 0.001), indicating a progressive increase in rooting percentages throughout the experimental period, regardless of treatment. Although the main effect of AC was not statistically significant (*p* = 0.107), a significant treatment × time interaction was observed (*p* < 0.001), revealing that the effect of activated charcoal on root induction depended on the duration of culture. These results indicate that activated charcoal did not accelerate early root induction but modified the temporal dynamics of rooting, enhancing root formation at later stages. This delayed effect is consistent with the ability of activated charcoal to adsorb inhibitory phenolic compounds and residual cytokinins that accumulate over time in the culture medium, a phenomenon previously reported in several *Rosa* species [34,35,36].

#### 2.3.2. Assay 2: Effect of Basal Medium, Auxin, and Photoperiod

**Figure 4:**
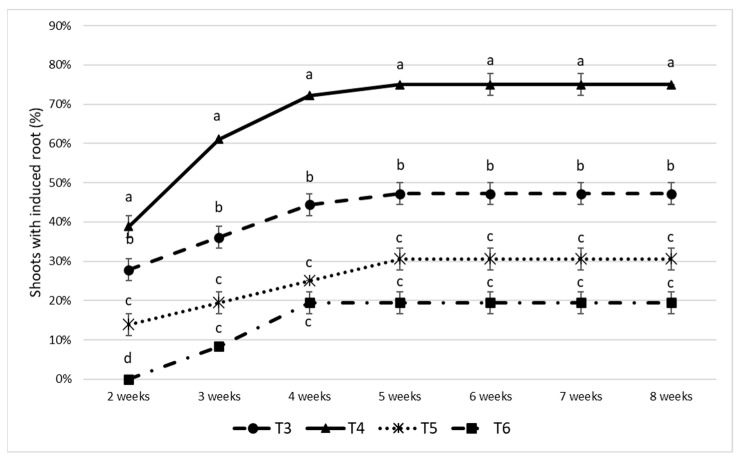
Time course of root induction (% shoots with induced roots) in *Rosa canina* under four culture conditions: WPM in darkness (T3) (●), WPM under a 16 h light/8 h dark photoperiod (T4) (▲), MS in darkness (T5) (■), and MS under a 16 h light/8 h dark photoperiod (T6) (×). Means with different letters, within each week, indicate significant differences among treatments (Tukey’s HSD, *p* < 0.05). Bars represent the SE.

To improve the rooting efficiency observed in the first assay, a second experiment (treatments T3–T6) was conducted incorporating indole-3-butyric acid (IBA) at 2 mg·L^−1^ and comparing two basal media (WPM and MS) under two photoperiod regimes (16 h light/8 h dark and continuous darkness). Mean values and SE for the accumulative percentage (%) of rooted shoots over time are shown in Figure 4.

In all treatments, root induction increased rapidly between weeks 3 and 5 and then plateaued, indicating the existence of a common induction window. Among the four treatments, the combination of WPM with IBA under a 16 h light/8 h dark photoperiod (T4) produced the highest and fastest rooting response, reaching 72–75% by weeks 4–5. WPM with IBA under continuous darkness (T3) achieved intermediate rooting levels, stabilizing at approximately 44–47%. In contrast, MS-based treatments were significantly less effective: MS with IBA under continuous darkness (T5) and MS with IBA under the light/dark photoperiod (T6) reached only about 31% and 19%, respectively.

The two-way ANOVA analysis revealed a highly significant main effect of basal medium composition on root induction (*p* < 0.00001), indicating that the choice of mineral formulation strongly influenced rooting performance. Photoperiod also had a significant effect (*p* = 0.00153), confirming that light conditions modulated adventitious root formation. Importantly, a highly significant basal medium × photoperiod interaction was detected (*p* < 0.00001), demonstrating that the effect of light on root induction depended on the basal medium used.

Compared with the auxin-free conditions of assay 1, the inclusion of the auxin IBA in assay 2 clearly enhanced both the speed and magnitude of root induction, particularly in WPM-based media. This result is consistent with previous studies demonstrating the central role of IBA in stimulating adventitious root formation in woody species [37]. In *R. canina*, in vitro experiments using 0.6–0.9 mg·L^−1^ IBA achieved 20–25% rooting, while auxin-free media yielded only 12% [21]. Ex vitro studies also confirm that auxin application improves rooting in cuttings [15,16,17].

The superior performance of WPM (T4 and T3) relative to MS (T5 and T6) in the presence of IBA confirms that basal medium composition plays a critical role in rhizogenesis. Similar findings were reported by Toma et al. [38], who observed faster root emergence and higher root numbers in *R. canina* explants cultured on WPM supplemented with low concentrations of IBA, whereas MS-based media consistently resulted in poorer rooting responses. These results support the notion that woody species often require nutrient formulations with lower ionic strength for optimal rhizogenesis.

The higher rooting percentage observed in treatment T4 compared with T3 (both WPM with IBA, but differing in photoperiod) suggests a possible interaction between auxin application and light exposure. In woody species, light has been shown to enhance adventitious root formation when combined with appropriate auxin levels—for example, in *Betula pendula*, maximum rooting and root density were achieved under light regimes at relatively low IBA concentrations, directly supporting this positive effect [39]. A testable hypothesis is that light perceived by photoreceptors may modulate root physiology and development in *R. canina*, influencing auxin transport and sensitivity [40]. Recent works on woody plant development and in vitro regeneration indicate that light intensity, spectrum, and photoperiod could regulate adventitious root formation, integrating photomorphogenic signaling with hormonal control [23,24], consistent with the superior performance of WPM + IBA under light photoperiod, as observed herein.

In contrast, no differences in rooting percentage were observed between photoperiod treatments when rooting was performed on MS medium with IBA (T5 and T6). This suggests that the promotive effect of light on root induction is expressed only when the basal medium provides a favorable physiological environment for auxin responsiveness. A reasonable hypothesis is that, under WPM, a 16 h light/8 h dark photoperiod resulted in a clear increase in rooting, whereas in MS medium, this response was lacking. This might be due to its higher salt concentration and nutrient composition, which may limit auxin-driven rhizogenesis. As a result, the positive effect of light becomes evident in WPM but remains undetectable in MS medium. This aspect represents a pending line of research that we intend to address in future experiments with *R. canina*, in order to determine whether a lower nutrient availability may enhance adventitious root formation. Similar nutrient-dependent restrictions have been documented in other re-calcitrant woody species, such as walnut [41] and pear [42], where the basal medium had to be reduced to one-fourth or one-half the macronutrient concentration to favor root induction and elongation.

To our knowledge, few studies have simultaneously evaluated rooting responses under MS and WPM media while also comparing light versus darkness conditions. The present results indicate that light enhances root induction in *R. canina* only when shoots were cultured with WPM, suggesting a medium-dependent photoperiod effect, which constitutes a key conceptual contribution of this work. Although the physiological basis underlying this response was not investigated here, differences between MS and WPM media—particularly their higher ionic strength and higher nitrogen content ratios in MS—have been reported to influence auxin metabolism in woody species [37,38,43,44,45]. This interaction between mineral nutrition, hormonal regulation, and light signaling represents a promising avenue for further research aimed at improving rooting efficiency in woody species that are traditionally considered difficult to propagate in vitro.

### 2.4. Root Development

**Figure 5:**
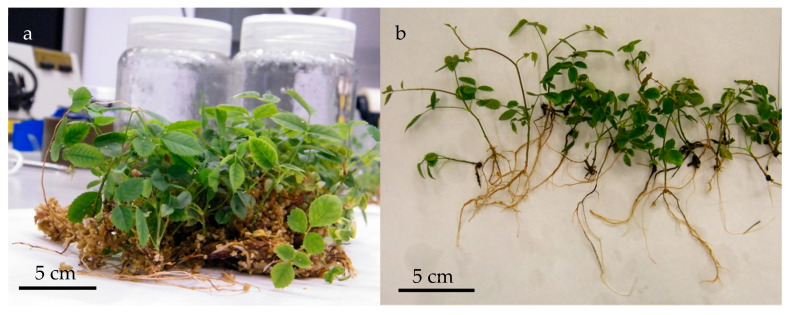
Shoots of *Rosa canina* removed from the jars containing the rooting medium, before (**a**) and after (**b**) being washed with water.

Shoots that successfully formed adventitious roots during the induction phase were transferred to a root development medium to promote root elongation and maturation. Under these conditions, induced primary roots elongated rapidly and gave rise to numerous secondary roots, resulting in well-structured and highly branched root systems (Figure 5).

The root development medium consisted of WPM basal salts combined with vermiculite, which provided a porous substrate with high aeration and drainage capacity. Adequate oxygen availability is a critical factor during root growth, particularly in woody species, where hypoxic conditions can severely impair rhizogenesis and root functionality. Previous studies in roses have demonstrated that insufficient aeration negatively affects root elongation and branching, whereas improved oxygen diffusion enhances both root growth and post-transplant performance [46,47]. Rooting of other woody species has been favored by adding vermiculite, such as apple rootstocks and walnuts [41,48,49], pears [42], chestnuts [50], hybrid tea roses [51], and several ornamental *Prunus* spp. [52], and for peach and nectarine embryo rescue [53,54,55,56].

Incorporation of vermiculite into the culture system likely improved gas exchange at the root–medium interface, facilitating respiratory activity and supporting sustained root growth [55]. As a result, roots produced during this phase were robust, elongated, and morphologically suitable for ex vitro transfer, indicating that the transition from a solid agar-based induction medium to a more aerated development substrate is a critical step for successful micropropagation of *R. canina*.

### 2.5. Acclimatization of Plantlets

The presence of numerous secondary roots, ranging from 5 to 15 cm in length, was consistently observed on plantlets after culture in vermiculite-containing medium, constituting a key factor for successful acclimatization. A well-developed and branched root system enhances water and nutrient uptake during the transition from heterotrophic in vitro conditions to autotrophic growth under greenhouse environments.

**Figure 6:**
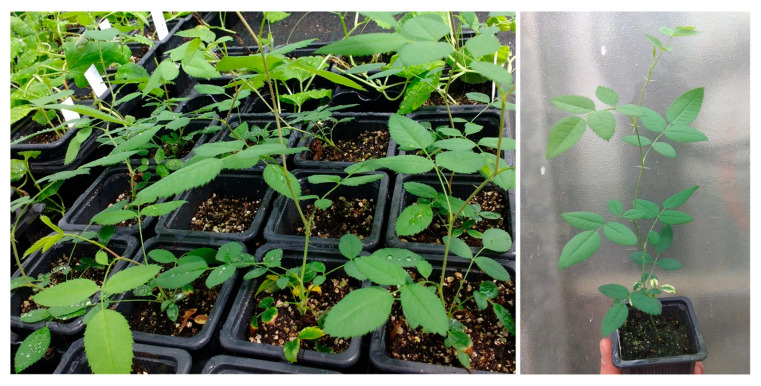
Plants of *Rosa canina* acclimated in the greenhouse.

Once the plantlets were transferred to the greenhouse and the acclimation cycle was completed, the acclimated plants exhibited normal morphology, active vegetative growth, and a 100% survival rate. These results demonstrate that the in vitro-derived root systems were fully functional and capable of supporting ex vitro development and are consistent with those obtained in a previous study on the acclimatization of *R. canina* plantlets, in which survival rates of 95–100% were achieved [57]. The successful acclimatization of all plants confirms the robustness of the micropropagation protocol and its suitability for the large-scale production of *R. canina* plants from wild germplasm. After initial acclimatization, plants were relocated to mist-controlled benches, where they continued to harden and develop under standard greenhouse conditions (Figure 6).

**Figure 7:**
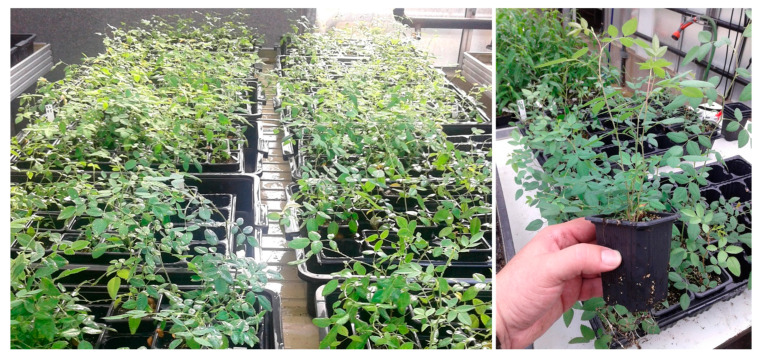
Greenhouse-hardened *Rosa canina* plants prepared for field transplanting.

The plants continued to grow and harden under greenhouse conditions until final delivery (Figure 7), where they were directly transplanted into commercial field plots.

## 3. Materials and Methods

### 3.1. Plant Material and Explant Establishment

**Figure 8:**
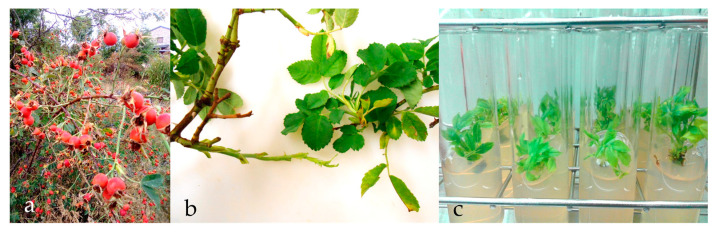
Wild *Rosa canina* plants collected in the field (**a**); branches used as the source of explants (**b**); introduced explants (**c**).

Branches of *Rosa canina* L. were collected from wild plants (Figure 8a) provided by Gratacool from La Cerdanya (Lleida, Spain). Axillary wood buds were excised (Figure 8b) and surface-disinfected by immersion in 70% (*v*/*v*) ethanol for 1 min, followed by a solution of 0.5% (*v*/*v*) NaOCl + Tween 80 for 15 min. Explants were rinsed with distilled sterile water for 3 times under aseptic conditions and introduced into tubes containing 15 mL of Murashige and Skoog (MS) basal medium [58] (Duchefa Biochemie B.V, Haarlem, The Netherlands) with 30 g·L^−1^ sucrose (Duchefa Biochemie B.V, Haarlem, The Netherlands), 6-benzylaminopurine (BAP) 1 mg·L^−1^ (Duchefa Biochemie B.V, Haarlem, The Netherlands), and 0.95% (*w*/*v*) agar (Quimivita, Barcelona, Spain) (Figure 8c). pH was adjusted to 5.70 prior to autoclaving.

Unless otherwise stated, all cultures were maintained at 22 °C under a 16 h light/8 h dark photoperiod with a light intensity of 150–200 μmol m^−2^·s^−1^.

Explants were transferred weekly to fresh medium, and the basal portion was excised to reduce phenolic accumulation and promote shoot development.

### 3.2. Shoot Propagation and Elongation

Newly formed shoots, when they reached 1–1.5 cm long, were excised and introduced into culture jars with a total capacity of 700 mL, each containing 100 mL of propagation medium. The multiplication phase was conducted in two sequential subcultures. In the first subculture, shoots were grown on MS basal medium with 30 g·L^−1^ sucrose, 2 mg·L^−1^ BAP and 0.95% (*w*/*v*) agar. After 3 weeks, shoots were separated in aseptic conditions and transferred to the same medium containing a reduced BAP concentration (1 mg·L^−1^).

Three weeks later, 50 mL of liquid MS medium with 30 g·L^−1^ sucrose, activated carbon 3 g·L^−1^, but without plant growth regulators, was added to each jar. Shoots were maintained under the same environmental conditions for an additional 3 weeks.

### 3.3. Root Induction Experiments

Elongated shoots (≥3 cm) obtained from the previous phase were selected for rooting experiments. Basal leaves were removed and shoots were individually transferred to sterile glass tubes containing the respective rooting media.

Two independent assays were carried out to evaluate the effects of basal medium composition, activated charcoal, auxin supplementation, and photoperiod on *R. canina* root induction.

#### 3.3.1. Assay 1: Effect of Activated Charcoal

Shoots were cultured on Woody Plant Medium (WPM) either without activated charcoal (T1) or supplemented with 3 g·L^−1^ activated charcoal (T2), both without auxin. Cultures were maintained under a 16 h light/8 h dark photoperiod (Table 2).

#### 3.3.2. Assay 2: Effect of Basal Medium, Auxin and Photoperiod

In the second assay, the effect of auxin addition was evaluated by incorporating indole-3-butyric acid (IBA) at a concentration of 2 mg·L^−1^ into the medium. Two basal media (WPM and MS) were compared, and two photoperiod regimes (16 h light/8 h dark and continuous darkness) were tested, resulting in four treatments (T3–T6) (Table 3).

All rooting media contained 30 g·L^−1^ sucrose and 0.95% agar, and the pH was adjusted to 5.70 before autoclaving.

Each treatment consisted of three replicates with 12 shoots per replicate (n = 36). Cultures were maintained under controlled conditions at 22 °C, and root induction was evaluated weekly from 2 up to 8 weeks.

All culture media were prepared using Murashige and Skoog (MS) or Woody Plant Medium (WPM) basal salts, plant growth regulators (BAP and IBA), and activated charcoal, provided by Duchefa Biochemie (Haarlem, The Netherlands).

### 3.4. Root Development

Shoots that formed adventitious roots were transferred to a root development medium, consisting of 40 mL of WPM basal medium (Duchefa Biochemie, Haarlem, The Netherlands) with 30 g·L^−1^ sucrose, 0.6% (*w*/*v*) agar combined with 50 mL of vermiculite per jar. Cultures were maintained for 4 weeks under the same environmental conditions described above.

### 3.5. Acclimatization

Plantlets exhibiting well-developed root systems and achieving a minimum shoot height of 5 cm were selected for acclimatization. Plantlets were carefully removed from the culture vessels and thoroughly rinsed under running tap water to eliminate residual vermiculite and culture medium (Figure 5). During this step, meticulous removal of the substrate was essential to prevent mechanical damage to the roots while ensuring complete elimination of residual sugars, as these compounds can serve as substrates for fungal colonization during acclimatization.

Immediately after washing, the plantlets were transplanted into trays containing a peat–vermiculite substrate (2:1, *v*/*v*); the peat used was the Exclusive brand (Gebr. Brill Substrate GmbH & Co. KG, Georgsdorf, Germany). Trays were placed inside plastic humidity-controlled tunnels located in the greenhouse, where relative humidity was gradually reduced from approximately 100% to 60% over a period of 14–21 days. These tunnels maintained soil temperatures above 22/18 °C (day/night) and provided a 16 h photoperiod, supplemented with LED lighting (SUP12100DC, AlternativaLED, Terrassa, Barcelona, Spain) to extend daylight hours, delivering 230 μmol m^−2^·s^−1^ photosynthetically active radiation (PAR) at the leaf level, promoting a progressive adjustment of the stomatal apparatus and cuticular development

After completing acclimatization, trays were moved to greenhouse benches, and the plants were irrigated using a misting system until they were suitable for field establishment.

### 3.6. Experimental Design and Statistical Analysis

All experiments were conducted using a completely randomized design. For each treatment, three independent replicates were established, each consisting of 12 explants (n = 36 per treatment). Rooting percentages were recorded weekly from 2 to 8 weeks.

For the first assay (T1–T2), data were analyzed using a two-way analysis of variance (ANOVA) with treatment (WPM vs. WPM + activated charcoal) and time (weeks) as fixed factors. The interaction between treatment and time was included in the model to evaluate whether rooting dynamics differed between treatments over time. When significant effects were detected, mean comparisons were performed using Tukey’s honestly significant difference (HSD) post hoc test. All statistical analyses were conducted at a significance level of α = 0.05. Data are presented as mean ± standard error (SE).

For the second assay (T3–T6), a two-way factorial ANOVA was performed to evaluate the effects of basal medium (MS vs. WPM), photoperiod (16 h light/8 h dark vs. continuous darkness), and their interaction on root induction. When significant effects were detected, means were compared using Tukey’s HSD post hoc test. All statistical analyses were conducted at a significance level of α = 0.05. Data are presented as mean ± standard error (SE).

Before conducting the ANOVA, residuals were examined to verify normality using the analytical tools available in JMP (JMP Student Edition version 18.2.2., SAS Institute Inc., Cary, NC, USA).

## 4. Conclusions

The present study establishes a complete, reproducible, and efficient in vitro micropropagation protocol for *Rosa canina* L., starting from wild explant introduction and culminating in successful ex vitro acclimatization. The entire process can be completed in six months. During the establishment stage (4 weeks), subculturing weekly to fresh medium is enough for controlling phenolics. The two sequential cytokinin treatments in the multiplication phase (3 weeks + 3 weeks) promote the production of vigorous shoots. The elongation step (3 weeks) proves essential for obtaining shoots of sufficient length and physiological quality for rooting. Using the combination of WPM, IBA, and a 16 h light/8 h dark photoperiod results in the fastest and highest rooting percentages (72–75% in 4–5 weeks). The transfer of rooted shoots to an aerated root development system incorporating vermiculite (4 weeks) promotes root elongation and extensive secondary root formation, generating robust and functional root systems. These roots enable plantlets to achieve high survival rates (100%) and normal growth when transferred to greenhouse conditions for the acclimatization step (3 weeks).

Successful rhizogenesis in *R. canina* is not governed by auxin alone but instead depends on the coordinated interaction of basal medium composition, auxin availability, and photoperiod: the same concentration of IBA can lead to markedly different outcomes depending on the ionic strength and nitrogen profile of the medium, as well as on the prevailing light regime. This interdependence indicates that future research should move beyond the traditional approach of testing isolated variables—such as a fixed photoperiod, a single auxin concentration, or a limited set of basal media—and instead adopt experimental designs that explicitly explore the full factorial combinations of these factors. Recognizing that medium composition, hormonal context, and light conditions can modulate each other’s effects opens the door to broader, multi-parameter studies aimed at identifying species-specific optima and understanding how these interacting signals collectively shape the rooting response in woody species.

## Figures and Tables

**Table 1:** Accumulative percentage (%) of rooted shoots of *Rosa canina* over time in WPM medium with or without activated charcoal (AC).

	Rooting Treatment			
	T1		T2	
Weeks	Mean	SE	Mean	SE
**2**	22.2 a *	2.8	16.7 a	0.0
**3**	25.0 a	0.0	16.7 b	0.0
**4**	25.0 a	0.0	16.7 b	0.0
**5**	25.0 a	0.0	16.7 b	0.0
**6**	30.6 a	2.8	16.7 b	0.0
**7**	36.1 a	2.8	50.0 a	4.8
**8**	41.7 b	0.0	58.3 a	4.8

* Treatment means with different letters indicate significant differences (Tukey’s HSD, *p* < 0.05). SE = Standard Error.

**Table 2:** Media composition and photoperiod conditions for root formation induction in *Rosa canina* shoots in Assay 1.

Treatment	Basal Medium	Additional Compounds	Photoperiod Conditions
**T1**	WPM	None	16 h light/8 h dark
**T2**	WPM	Activated charcoal (3 g·L^−1^)	16 h light/8 h dark

**Table 3:** Media composition, plant growth regulators (PGRs) and photoperiod conditions for root formation induction in *Rosa canina* shoots in Assay 2.

Treatment	Basal Medium	PGRs	Photoperiod Conditions
**T3**	WPM	IBA (2 mg·L^−1^)	Continuous darkness (24 h)
**T4**	WPM	IBA (2 mg·L^−1^)	16 h light/8 h dark
**T5**	MS	IBA (2 mg·L^−1^)	Continuous darkness (24 h)
**T6**	MS	IBA (2 mg·L^−1^)	16 h light/8 h dark

## Data Availability

Data will be made available upon request.

## Abbreviations

The following abbreviations are used in this manuscript:

MS	Murashige and Skoog
WPM	Woody Plant Medium
PGR	Plant Growth Regulator
BAP	6-benzylaminopurine
IBA	Indole-3-butyric acid
GA3	Gibberellic acid
NAA	Naphthaleneacetic acid
AC	Activated charcoal
